# A Review: Proteomics in Nasopharyngeal Carcinoma

**DOI:** 10.3390/ijms160715497

**Published:** 2015-07-08

**Authors:** Ze-Tan Chen, Zhong-Guo Liang, Xiao-Dong Zhu

**Affiliations:** 1 Department of Radiation Oncology, Cancer Hospital of Guangxi Medical University and Cancer Institute of Guangxi Zhuang Autonomous Region, Nanning 530021, China; E-Mails: chenzetan@163.com (Z.-T.C.); zhongguoliang1989@126.com (Z.-G.L.); 2 Key Laboratories of High-Incidence-Tumour Prevention and Treatment, Guangxi Medical University, Ministry of Education, Nanning 530021, China

**Keywords:** nasopharyngeal carcinoma, NPC (nasopharyngeal carcinoma), proteomics, two-dimensional electrophoresis, mass spectrometry, biomarker, differentially expressed proteins

## Abstract

Although radiotherapy is generally effective in the treatment of major nasopharyngeal carcinoma (NPC), this treatment still makes approximately 20% of patients radioresistant. Therefore, the identification of blood or biopsy biomarkers that can predict the treatment response to radioresistance and that can diagnosis early stages of NPC would be highly useful to improve this situation. Proteomics is widely used in NPC for searching biomarkers and comparing differentially expressed proteins. In this review, an overview of proteomics with different samples related to NPC and common proteomics methods was made. In conclusion, identical proteins are sorted as follows: Keratin is ranked the highest followed by such proteins as annexin, heat shock protein, 14-3-3σ, nm-23 protein, cathepsin, heterogeneous nuclear ribonucleoproteins, enolase, triosephosphate isomerase, stathmin, prohibitin, and vimentin. This ranking indicates that these proteins may be NPC-related proteins and have potential value for further studies.

## 1. Introduction

Nasopharyngeal carcinoma (NPC) is an endemic disease that is epidemic in areas of Southeast Asia, Southern China, Hong Kong, and Taiwan [1]. In 1978, the incidence of this disease in Southeast Asia was approximately 15–50/100,000 men per year [2]. In 2010, the data showed that the incidence and mortality rates in Southern China were 19.5, including Hong Kong, with 7.7 per 100,000 persons [3]. So far, the etiology of NPC includes genetic components, such as aberrations in chromosomes 1, 3, 9, 11, 12, and 14; infectious factors, such as Epstein-Barr virus (EBV); and environmental elements, such as the consumption of salt-preserved fish [4,5]. According to the (World Health Organization) WHO classification, the pathological type of NPC is classified into keratinizing squamous cell carcinoma (type I, known as well differentiated), differentiated nonkeratinizing carcinoma (type II, known as moderately differentiated), and undifferentiated carcinoma (type III, known as poorly differentiated) [6]. In fact, in the areas mentioned above with high incidence, at least 95% of NPC patients are of the poorly differentiated (WHO types II and III) pathological type and are thus sensitive to radiotherapy and chemotherapy [7]. Subsequently, radiotherapy and chemotherapy are the main treatments for NPC [8]. However, over several decades, changes in different fields, such as advances in radiotherapy techniques that evolved from 2-dimensional radiotherapy to 3-dimensional conformal techniques and then to intensity modulated techniques, dose escalation, and using concurrent cisplatin-based chemotherapy; unfortunately, approximately 20% of NPC patients still have local recurrence with the above therapeutic schemes [9,10,11]. In addition, a well-known cause of local recurrence with poor survival in NPC is radioresistance [12]. Recently, a large body of research showed that radioresistance was related to inducing pathways (e.g., the PI3K/Akt pathway [13], NF-κB pathway [14], and Wnt pathway [15]), altering molecules (e.g., high mobility group protein box 1 (HMGB1) [16], metadherin (MTDH) [17], and microRNA [18]), mediating tumour anoxic effect [19], facilitating the tumour angiogenesis [20], cancer stem cells [21] and autophagy [22]. In addition, many studies have demonstrated that the biological behaviour and prognosis of NPC patients could be significantly different in patients with the same stage, histologic type, or differentiation grade, implying that the existence of other. Taken together, there is a gap in studying the radioresistance and factors related to the behaviour an factors, such as molecular variables and ethnicity, can affect the behaviour and prognosis of NPC [23] prognosis of NPC.

Currently, due to high-throughput techniques, such as GeneChip, protein-chip and OMICS-analysis, a large number of studies have been conducted to determine the mechanism underlying the aetiologies and prognosis. In this paper, we will provide a review of proteomics and nasopharyngeal carcinoma, illustrate the progress in proteomics, and summarize molecules that are identical in different studies.

## 2. The Development of Proteomics Technologies

The term “proteome” refers to the total protein complement of a genome, of which the genome refers to the genetic requirements for life with the potential to express proteins [24]. The fundamental and developmental stages of proteomics depend on technologies for protein separation and identification. For protein separation and visualization technologies, one-dimensional polyacrylamide gel electrophoresis (1-DE), two-dimensional polyacrylamide gel electrophoresis (2-DE), differential gel electrophoresis (DIGE), isotope-coded affinity tags (ICATs), stable isotope labelling of amino acids (SILAC), and isobaric tags for relative absolute quantitation (iTRAQ) are developing and growing gradually. For protein identification technologies, Matrix-Assisted Laser Desorption/Ionization Time of Flight Mass-Spectrometry (MALDI-TOF MS), liquid chromatography-electrospray ionization-mass spectrometry, and surface-enhanced laser desorption ionization time of flight mass spectrometer (SELDI-TOF MS) are widely used.

### 2.1. Protein Separation and Visualization Technologies

**1-DE**. One-dimensional SDS-polyacrylamide gel electrophoresis (1-D SDS-PAGE, 1-DE) is used for molecule separation by size and charge, of which SDS is a detergent that denatures second and nondisulphide-linked tertiary structures and combines them with a negative charge that correlates with their length, enabling molecular weights to be estimated [25]. SDS is a non-continuous gel with an upper stacking gel and lower resolving gel that have different pH values and polyacrylamide concentrations. In addition, small proteins will move through the polyacrylamide gel more quickly than large proteins. In fact, the percentages of polyacrylamide can be optimized for the size range of molecules that are present in the sample [26]. Based on the percentages of polyacrylamide and the characteristics of each protein, the proteins are separated in bands.

**2-DE**. Based on 1-DE technology, 2-D SDS-PAGE (2-DE) was established and developed [27]. 2-DE consists mainly of two steps of separation: the first and the second dimensions. In the first dimension, protein molecules are resolved depending on their molecular weight (*M*_W_) and charge known as the isoelectric point (pI). In the second dimension, protein separation is performed based on the *M*_W_. Because different protein molecules have different *M*_W_ or/and PI, proteins are efficiently separated by 2-DE rather than by 1-DE [27]. In addition, a modified method called immobilized pH gradients (IPG) was used in 1991 for the first-dimension separation of 2D PAGE, in which the pH separation range could be defined from broad to extremely narrow [28]. Because of its advantages in robustness, visualized mapping analysis, and having a compatible platform for further analysis, 2-DE is a powerful tool that is used for the separation and fractionation of protein mixtures from humans, animals, plants, or other microorganisms, becoming a more popular and comprehensive technology that is used to simultaneously fractionate, identify, and quantify proteins when combined with mass spectrometric (MS) identification or other immunological tests [29].

**2-DIGE** (two-dimensional difference gel electrophoresis). However, 2-DE has its own disadvantages, such as low reproducibility [30], difficulty in separating hydrophobic and extremely acidic or basic proteins [31], and narrow dynamic range and is low-throughput and labour-intensive [28]. Thus, a 2-DE modification system, such as representational 2-DIGE technology, emerged [32,33]. 2-DIGE is differential gel electrophoresis that circumvents these problems. The workflow of 2-DIGE is as follows: two protein samples are pre-labelled with two cyanine dyes (e.g., Cy3 and Cy5), thus enabling two different samples to run on the same gel in both dimensions. Hence, the two samples are exposed to the same procedure and environment throughout the experiment. Then, protein spots can be detected by fluorescence imaging after electrophoresis with a sensitivity that is equal to that of silver staining. Finally, differences in protein composition between the two samples due to differences expression of protein can be identified without any post-processing of the gel. Due to the use of an internal standard in every gel, overcoming the problem of inter-gel variation, and reducing the number of gels needed for each experiment, 2-DIGE efficiently provides the strict and reproducible differential expression values for proteins in more than two biological samples [34]. Popularly, 2-DIGE is one of the approaches that are applied to quantitative proteomics with great sensitivity and accuracy in quantitation.

Nevertheless, separation technologies of 2-DE and 2-DIGE are gel-based methods that have the typical problems of reducing enzyme accessibility to the protein; Missing large peptides that are extracted from the gel, leading to reduced protein coverage; and avoiding the need to identify potentially hundreds of individual spots [35]. However, some methods, such as (i) Pre-fractionation techniques able to reduce sample complexity; (ii) High-resolution and limited-range IEF (isoelectric focusing) improving protein resolution on gels; (iii) Protein fluorescent labelling, resulting in more accurate protein visualization; and (iv) Efficient detergents enhancing membrane protein separation, were improved [36]. Importantly, LIEF (liquid isoelectric focusing) is a technology that fractionates a complex mixture according to the pI in a no-gel medium, and the fractions can be assembled and directly analyzed by other techniques [37]. However, the improvements did not go far enough. Thus, other separation techniques coupled with MS, especially those that do not use gels, such as isotope-coded affinity tags (ICATs), stable isotope labelling of amino acids (SILAC), and iTRAQ, which are generally known as gel-free methods, were devised.

**ICATs** (isotope-coded affinity tags). As discussed by Steve *et al.* [38], the automated, data-dependent (electrospray-tandem mass spectrometry) ESI-MS/MS, combined with LC, which is one of the most common gel-free proteomics methods, and database searching significantly increase the sensitivity and speed for the identification of gel-separated proteins, and LC-MS/MS has been used successfully for the large-scale identification of proteins directly from mixtures without gel electrophoretic separation. However, these methods were not quantitative and were also incompatible with the analysis of low-abundance proteins. Thus, Aebersold’s group described a new approach for the concurrent sequence identification of the individual proteins within complex mixtures [39]. This approach was based on a class of new chemical reagents that were named isotope-coded affinity tags (ICATs) and tandem mass spectrometry, of which ICATs consist of three functional elements: specific chemical reactivity (“light” or “heavy” ICATs linking with cysteinyl residues of proteins), isotopically coded linkers, and affinity tags. In fact, any amount of starting material could theoretically be used with the ICAT scheme, and ICATs could be used to detect sufficient amounts of very low-abundance proteins by mass spectrometry. However, not all proteins contain cysteinyl residues, resulting in the inefficiency of ICATs to analyze these proteins [36]. Furthermore, this approach based on “light” or “heavy” ICATs can only be applied to comparative groups of two, but not of two more, and show a limitation to those of several experimental controls or time-course studies.

**SILAC**. The concept of SILAC (stable isotope labelling of amino acids) was originally described by Matthias’ group [40]. Research showed that mammalian cell lines that were grown in a non-radioactive, isotopically labelled form of essential amino acid instead of a standard amino medium rather than a normal medium, were not significantly different. It is well known that the essential amino acids include methionine, valine, lysine, isoleucine, phenylalanine, leucine, tryptophan, and threonine. Thus, essential amino acids are added to amino acid-deficient medium and then incorporated into all proteins as they are synthesized, followed by MS identification. For example, the SILAC labelling and combined fractional diagonal chromatography (COFRADIC) isolation of methionine-containing peptides allowed Andrea’s group to quantify differences in cancer-derived myofibroblasts compared to adjacent tissue-derived myofibroblasts, revealing an increased abundance of several proteases in cancer myofibroblasts, such as matrix metalloproteinases (MMP)-1 and -3 [41]. In addition, valine [42], lysine [43], isoleucine [44], phenylalanine [45], leucine [46], and tryptophan [47] were used in proteomics. Therefore, SILAC is a simple, inexpensive, and accurate process that can be used as a quantitative proteomic scheme in any cell culture system. In fact, this method overcomes the disadvantage of ICATs in quantitation changes in proteins that may not contain any cysteine residues at all. In addition, labelling uniformly, labelling specifically, and virtually 100% incorporation are advantages of SILAC. However, SILAC relies on living cells.

**iTRAQ**. iTRAQ is a multiplexed set of reagents for quantitative protein analysis that puts isobaric mass labels at the N termini and lysine side chains of peptides in a digestion mixture. According to reagent labelling, signature or reporter ions following CID are used to identify and quantify individual members of the multiplex set [48]. The isobaric nature of the labels permits the simultaneous comparison of multiple samples. Importantly, expression ratio measurement demonstrated high consistency, and peptide coverage is significantly increased relative to ICATs. The labelling chemistry is global in that any peptide with a free amine can be labelled and measured [48]. So far, 4-plex multiplex [48] and 8-plex multiplex [49] have been applied to this strategy, and it has complemented the 2-DE, 2DEIG, ICATs, and SILAC methods within proteome analyses.

### 2.2. Protein Identification Technologies

Since the theory behind mass spectrometry (MS) was put forward, identification technologies, such as Matrix-assisted laser desorption ionization (MALDI) mass spectrometry [50] with molecular masses exceeding the quantification of 10,000 Daltons and electrospray ionization (ESI) based on accurate mass data and the true isotopic pattern, have been developed [51,52]. Shortly afterwards, to solve the problem of measuring the composition of single aerosol particles, Thomson *et al.* [53] in 1992 undertook an effort to measure these thresholds as a function of particle composition, laser wavelength, and particle size with a time-of-flight mass spectrometer (TOF-MS). In 1995, the concept of “proteomics” was reported [24]. Thus, these methods were applied to peptide mapping with large amounts of sample and posttranslational modifications. In 1996, the first large-scale protein identification project was conducted and demonstrated definitively that mass spectrometry had the capacities of sensitivity, specificity and throughput for this task [54]. MALDI-MS profiling requires the solubilization of proteins/peptides, then removing the lipids, salts and other interfering compounds, following a very small volume of solubilized protein mixing, embedding and crystallizing with a matrix solution, and producing protein ions carrying a single charge for MS analysis [55]. For ESI-MS profiling, ESI generates charged droplets in a high-voltage field that produces multiply charged ions during subsequent Coulomb explosions. Although this approach is less stable, it is less susceptible to signal suppression [56]. Due to technology constantly evolving, not only water soluble proteins but also hydrophobic membrane-bound proteins were examined by MS [57].

A mass spectrometer includes three parts: ion source, mass analyzer, and detector. In fact, MALDI and electrospray ionization (ESI) are widely used for ion sources in biological research, while TOF is widely used for mass analyzers. In general, we can obtain a mass spectrum of proteins/peptides via MALDI-TOF-MS/ESI-TOF-MS. It is worth mentioning that a mass spectrum represents the relative abundances of ionizable molecules with various mass-to-charge (*m*/*z*) values, which range for MALDI-TOF-MS from several hundred *m*/*z* to a few tens of thousands *m*/*z*. Subsequently, an *m*/*z* value in MALDI mass spectrometry is interpreted as the molecular mass, as ions with a charge of +1 prevail. Consequently, an intensity of a spectrum at an *m*/*z*-value represents the relative abundance of a compound with this *m*/*z*-value. Although MALDI is not a quantitative technique, it is extensively used for semi-quantitative comparisons that rely on the relative abundance of molecules with a spectrum or, after the normalization of spectra, between spectra [58,59].

Surface-enhanced laser desorption/ionization time of flight mass spectrometry (SELDI-TOF-MS) was presented in 1993 for the first time [60]. Since then, many reports regarding the application of the SELDI-TOF-MS technology, such as the ProteinChip system, have been published. In fact, this technique is one type of modification of MALDI that uses a target that is modified to reach biochemical affinity together with the sample proteins. The workflow of SELDI-TOF-MS is that the mixture is placed on a surface that is modified with a chemical property, such as binding affinity. Then, the matrix is applied to the surface with the sample peptides for crystallization. In the steps of binding and washing off, surface-bound proteins are left for analyses. Finally, samples that are spotted onto a SELDI surface are typically analyzed with a TOF-MS and detector [61]. SELDI technology has some advantages, such as the mixture being deposited onto a surface without being dried over MALDI, making SELDI suitable for serum analysis, which contains a large amount of low-molecular-weight (<15 kDa) and low-abundance proteins that bring important diagnostic information [62]. Low-molecular-weight and low-abundance proteins exist below the detection limits of any conventional testing. In addition, SELDI-TOF-MS provides selective and rapid protein expression profiles from a variety of biological samples (e.g., antibodies, other proteins with proper binding properties, or even DNA) with minimal requirements for purification and separation prior to mass spectrometry [63,64]. Thus, this technology has become the most promising technology for the early detection of cancer markers to form a pattern with higher sensitivity and specificity [65,66,67]. In addition, SELDI-TOF-MS has also been applied to diseases such as pre-eclampsia, PCOS [68], and neural tube defects [69], for proteomic profiling.

## 3. Current Proteomic Research into NPC

Proteomic approaches are important and useful for studying the human proteome of NPC, including protein expression in one sample, differential protein expression between samples, post-translational modifications, protein-protein interactions and signalling pathways. Proteins of samples come from cell lines, NPC-bearing mice, and human tissue or fluid. In this review, proteomics with different samples related to NPC are described in chronological sequence.

### 3.1. Proteomics Research in the Cell Line of NPC

HNE1 (poorly differentiated nasopharyngeal carcinomas cell line), HNE2, HNE3, CNE-1 (human highly differentiated NPC cell line), CNE-2 (human poorly differentiated NPC cell line), SUNE1 (poor differentiation and high metastasis), 5-8F (high metastatic potential), 6-10B (without metastatic potential), S-18 (high-metastatic), NPC-TW02 (derived from a keratinizing carcinoma), NPC-TW04 (derived from an undifferentiated carcinoma), NPC-BM1 (derived from a bone marrow biopsy of a female Taiwanese patient with NPC), HONE-1 (poorly differentiated nasopharyngeal carcinomas), HK1 (well-differentiated squamous nasopharyngeal carcinomas), ATCC: HTB-43, and C666-1 are NPC cell lines, while NP69 is a normal nasopharyngeal cell line.

The proteomic profiling of NPC was first used by the Institute of Xiangya Medicine of China in 2002 [70]. As previously noted, chromosome 3 was related to NPC [2]. Moreover, NAG7 was cloned from the common minimal deletion region in 3p25.3–26.3 [71]. Therefore, to elucidate the mechanism of NAG7, the NAG7 vector was constructed and transfected into HNE1 with liposomes [70]. Then, 2-DE and MALDI-TOF-MS were used to construct HNE1 and control the comparison of different proteins. The results showed that nine proteins that were up-regulated and seven proteins that were down-regulated were used to construct HNE1 compared to the controls (Table 1), which are involved in cell cycling, transcription, regulation and apoptosis. This study concluded that NAG7 was a tumour suppression gene.

**Table 1:** The proteomics of cell lines related to nasopharyngeal carcinoma.

Year	Proteins	Description	Reference
2002	Sorting nexzn11, *etc.*, were up-regulated in HNE1 transfection with NAG7; Plasminogen, *etc.*, were down-regulated in HNE1 transfection with NAG7.	HNE1 transfection with NAG7 *vs.* HNE1 cells; 2-DE and MALDI-TOF-MS.	[70]
2004, 2005	Triosephosphate isomerase and 14-3-3σ were up-regulated in CNE-2 treated with TPA; Stathmin, *etc.*, were down-regulated in CNE-2 treated with TPA.	CNE-2 treated with TPA *vs.* The control; 2-DE and MALDI-TOF-MS.	[72,73]
2005	The representative proteins are fibronectin, Mac-2 binding protein (Mac-2 BP), and plasminogen activator inhibitor 1 (PAI-1).	NPC-TW02 and NPC-TW04 cells were used to screen biomarkers; 8%–14% SDS gels and MALDI-TOF-MS.	[74]
2006	HSP 27, 14-3-3σ, HSP 70, *etc.*, were upregulated in p53-knockdown CNE-2 cells; Eukaryotic translation initiation factor 4B (eIF4B), heterogeneous nuclear ribonuclear protein K (hnRNP K), *etc.*, were down-regulated in p53-knockdown CNE-2 cells.	p53 knockdown CNE-2 cells *vs.* the control; 2-DE, MALDI-TOF-MS and ESI-Q-TOF-MS.	[75]
2006	R-enolase, maspin, *etc.*, were up-regulated in HONE-1 and HK1 with cetuximab; HSP gp96 was down-regulated in HONE-1 and HK1 with cetuximab.	HONE-1 and HK1 with cetuximab *vs.* the controls; 2-DE and MALDI-TOF MS.	[76]
2006	Annexin-A2, HSP 27, stathmin, annexin-I, *etc.*, were up-regulated in CNE-1-LMP1 cells.	CNE-1-LMP1 cells *vs.* CNE-1 cells; 2-DE and MALDI-TOF MS.	[77]
2007	Nm-23-H1, 14-3-3σ, keratin-18, HSP 27, triosephosphate isomerase, *etc.*, were up-regulated in 5-8F cells; Heterogeneous nuclear ribonucleoproteins C1/C2, α-enolase, annexin-A1, *etc.*, were down-regulated in 5-8F cells.	5-8F *vs.* 6-10B; 2-DE and MALDI-TOF-MS.	[78]
2008	Adenosine triphosphate synthase chain was up-regulated in C666-1 cells; Annexin-II, annexin-V, profilin 1, *etc.*, were down-regulated in C666-1 cells.	C666-1 compared with NP69; 2-DE and MALDI-TOF-MS.	[79]
2009	Cathepsin L, cofilin-1, superoxide dismutase, *etc.*, were up-regulated in CNE-2 with TGF-α; 14-3-3σ, annexin-A5, peroxiredoxin-1, *etc.*, were down-regulated in CNE-2 with TGF-α.	Conditioned media of CNE-2 with TGF-α *vs.* CNE-2 without TGF-α; 2-DE and MALDI-TOF-MS.	[80]
2009	Alpha-enolase, annexin-A1, L-lactate dehydrogenase B chain, triosephosphate isomerase, *etc.*	Secretory proteins of NPC-TW04; 12% SDS-PAGE and HPLC-MS/MS.	[81]
2009	Annexin-I was up-regulated in CNE-2-CDDP cells.	CNE-2-CDDP cells *vs.* CNE-2 cells; 2-DE and MALDI-TOF-MS.	[82]
2010	Cathepsin L1 and interferon-induced 17-kDa protein.	Secretory proteins of NPC-TW02, NPC-TW04, and NPC-BM1 cells; 8%–14% SDS-PAGE and LC-MS/MS.	[83]
2010	Cathepsin B, cofilin-1, profilin-1, l-Lactate dehydrogenase A chain, 14-3-3σ, heat shock cognate 71 kDa, cathepsin C, stathmin, manganese superoxide dismutase (MnSOD), *etc.*	NPC-TW02, NPC-TW04, and NPC-BM1 of secretory proteins; 8%–14% SDS-PAGE and LC-MS/MS.	[84]
2010	MnSOD were up-regulated in CNE-2-IR cells; 14-3-3σ and maspin were down-regulate in CNE-2-IR cells.	CNE-2-IR cells *vs.* CNE-2; 2-DE and MALDI-TOF-MA.	[85]
2010	Annexin-A3, Keratin, Glutathione *S*-transferase P, Heat shock protein β-1, Heterogeneous nuclear ribonucleoprotein K, HSP 60, Heterogeneous nuclear ribonucleoproteins C1/C2, Keratin-18, *etc.*, were up-regulated in CNE-2 with TGF-α; Vimentin, HSP 70, *etc.*, were down-regulated in CNE-2 with TGF-α.	Conditioned media of CNE-2 with TGF-α *vs.* CNE-2 without TGF-α; 2-DE and MALDI-TOF-MS.	[86]
2010	14-3-3σ, annexin-A3, annexin-A1, Heat-shock protein 105 kDa, Profilin-2, annexin-A5, *etc.*, were up-regulated in PRA1-knockdown NPC cells; Protein S100-A4, Poly [ADP-ribose] polymerase 1, *etc.*, were down-regulated in PRA1-knockdown NPC cells.	PRA1-knockdown NPC cells *vs.* the control; iTRAQ labelling approaches and LC-MS/MS.	[87]
2010	14-3-3σ, EGFR, keratin-8, and p53, *etc.*	Proteins of 5-8F cells related to cancer invasion and metastasis; SDS-PAGE and ESI-Q-TOF-MS.	[88]
2011	Keratin-8, Vimentin, Heterogeneous nuclear ribonucleoprotein K, HSP 60, Heterogeneous nuclear ribonucleoproteins C1/C2, annexin-A3, Glutathione *S*-transferase P1, Heat shock protein β-1, Stathmin, *etc.*, were up-regulated in CNE-2 treated with EGF; Heat shock cognate 71 kDa protein, Keratin-18, and Peroxiredoxin-2 were down-regulated in CNE-2 treated with EGF.	CNE-2 treated with EGF *vs.* the control; 2-DIGE and MALDI-TOF-MS.	[89]
2011	HSP 70, α-enolase, Heterogeneous nuclear ribonucleoproteins E2, annexin-A1, 14-3-3σ, annexin-A2, Prohibitin, peroxiredoxin-2, Glutathione *S*-transferase P1, and triosephosphate isomerase were up-regulated, while keratin-18 and keratin-8 were down-regulated in CNE-2/pSUPER with irradiation; HSP 70, annexin-A2, Heterogeneous nuclear ribonucleoproteins E2, NM-23-H1, triosephosphate isomerase, *etc.*, were up-regulated, while keratin-8 was down-regulated in CNE-2/sip53 with irradiation.	Irradiated CNE-2/pSUPER (CNE-2 transfected by empty vector) *vs.* Unirradiated control; Irradiated CNE-2/sip53 (p53 knockdown) compared with unirradiated control; 2-DE and MALDI-TOF-MS.	[90]
2011	Keratin-75, l-lactate dehydrogenase B chain, Triosephosphate isomerase, *etc.*, were up-regulated.	HTB-43 cells infected by EBV *vs.* the control; iTRAQ, 2-DE, and LC-MS/MS.	[91]
2011	Keratin-8, and HSP 27 were up-regulated in S-18 cells; HSP 70, keratin-18, and HSP 60 were down-regulated in S-18 cells.	S-18 *vs.* CNE-2 cells; 2-DE and MALDI-TOF-MS.	[11]
2012	Periostin, Keratin-8, annexin-A2, Heterogeneous nuclear ribonucleoprotein A3, HSP 27, *etc.*, were up-regulated in CNE-1-IR cells; Prohibitin, Heterogeneous nuclear ribonucleoprotein A1, *etc.*, were down-regulated in CNE-1-IR cells.	CNE-1-IR *vs.* CNE-1; 2-DE and MALDI-TOF-MS.	[92]
2012	Peroxiredoxin 3, peroxiredoxin 6, superoxide dismutase, prohibitin, *etc.*, were up-regulated in 5-8F cells; Maspin, *etc.*, were down-regulated in 5-8F cells.	5-8F *vs.* 6-10B cells; 2D-DIGE, MALDI-TOF PMF (peptide mass fingerprint), and ESI-Q-TOF MS/MS.	[93]
2012	Cofilin 1, *etc.*, were up-regulated in NESG1-overexpressing of 2F4, 3D8 cells; HSP 70, Heat shock protein 90 kDa β (Grp94), Keratin-8, Enolase 1 (α), Heterogeneous nuclear ribonucleoprotein H1 (H), Vimentin, Cathepsin D, *etc.*, were down-regulated in NESG1-overexpressing of 2F4, 3D8 cells.	NESG1-overexpressing of 2F4, 3D8 cells *vs.* the control of C6 cells; 2-DE and MALDI-TOF-TOF.	[94]
2012	Cathepsin D, Heat shock protein HSP 90-α, Keratin-1, annexin-A5, Heterogeneous nuclear ribonucleoproteins C1/C2, α-enolase of α-enolase, *etc.*, were up-regulated in CNE-2/cDDP cells; Keratin-18, NM-23, Heat shock protein β-1, *etc.*, were down-regulated in CNE-2/cDDP cells.	CNE-2/cDDP *vs.* CNE-2; 2-DE and ESI-Q-TOF-MS.	[95]
2012	End binding protein 1 was upregulated in HK1/ASC cells treated with EBER, H_2_O_2_, and poly (dA:dT).	ASC-expressing HK1 (HK1/ASC) cells treated with EBER, H_2_O_2_, and poly (dA:dT) *vs.* the controls; iTRAQ-LC-MS/MS.	[96]
2012	Cathepsin, cathepsin B, *etc.*, were up-regulated in 6-10B treat with DNP.	6-10B treat with DNP *vs.* The control; SILAC, 10% SDS-PAGE and LC-MS/MS.	[97,98]
2013	Galectin-1, Stathmin, Nm-23 protein, *etc.*, were up-regulated in CNE-2R cells; Annexin-A3, *etc.*, were down-regulated in CNE-2R cells.	CNE-2R *vs.* CNE-2 cells; 2-DE and MALDI-TOF-MS.	[99]
2013	U6 snRNA-associated Sm-like protein LSm2, Cytochrome c, 10 kDa heat shock protein mitochondrial, *etc.*	Secretome of NPC-TW02, NPC-TW04, and NPC-BM1 cells; Tricine-SDS-gel-assisted fractionation and LC-MS/MS.	[100]
2013	Cleaved PARP, caspase-3, and -12 were upregulated.	CNE-1 cells treated with isodeoxyelephantopin *vs.* the control. SILAC, 10% SDS-PAGE, and LC-MS/MS.	[101]
2013	14-3-3γ was up-regulated in CNE-1/CR1^+^ cells.	CNE-1/CR1^+^ *vs.* the parental cells; 2D-DIGE MALDI-TOF MS.	[102]
2014	Ferritin, *etc.*, were up-regulated in 6-10B treated with DNP; Eukaryotic translation initiation factor 4B, triosephosphate isomerase, *etc.*, were down-regulated in 6-10B treated with DNP.	6-10B treated with DNP *vs.* The control; SILAC and SCX-LC–MS/MS.	[103]

In 2004, the differentially expressed proteins between CNE-2 treated with 12-*O*-tetradecanoylphorbol 13-Acetate (TPA) and CNE-2 were analyzed by 2-DE and MALDI-TOF-MS. TPA is a tumour promoter and differentiation revulsant drug [72,73]. This result is mainly because at least 95% of NPC patient tumors are amazingly poorly differentiated, and TPA could induce CNE-2 cells to growth arrest and then from differentiation to death [7]. The differentially expressed proteins between the two above groups are displayed in Table 1. This study concluded that the 14-3-3σ protein was responsible for cellular differentiation and death, the decrease in nucleophosmin and stathmin related to growth arrest and apoptosis, and some morphological changes due to the lack of stathmin and Reticulocalbin 1 precursor.

To date, the available biomarkers for the early detection of NPC are EBV-associated proteins, which often present low sensitivity and specificity [104]. The diagnoses of NPC mainly depend on the endoscopic examination and histological observation of biopsies and often present later-stage disease. Therefore, biomarkers for early detection and prognosis would greatly improve NPC treatment and outcomes. In 2005, NPC-TW02 and NPC-TW04 were applied to search for biomarkers in serum-free medium [74]. The three proteins fibronectin, Mac-2 BP, and PAI-1 were expressed in serum-free medium, and triplets of them were also highly expressed in 46 NPC patients compared to 47 healthy controls. In addition, these above three proteins were expressed in nude mice that were inoculated with NPC-TW02 cells but either not or less expressed in normal nasopharyngeal tissues. This result indicates that the above-mentioned methods could be applied to other cell lines for searching biomarkers or other cancers.

P53 is a tumour-suppressor protein that is interestingly overexpressed in NPC [105]. In 2006, to detect proteins that were associated with the function of p53 in NPC, proteins from the p53 knockdown human NPC CNE-2 cell line were compared with the control in high-throughput screening by MALDI-TOF-MS and electrospray ionization-quadruple time-of-flight mass spectrometry (ESI-Q-TOF-MS) [75]. Subsequently, 22 proteins were differentially expressed between the two cell lines, and some of the differentially expressed proteins were associated with the p53 function (HSP27, heterogeneous nuclear ribonuclear protein K, 14-3-3σ, *etc.*), while others may be novel proteins that are related to p53 function (eukaryotic translation initiation factor 4B, tumour protein translationally controlled 1, *etc.*). Meanwhile, the overexpression of EGFR in NPC [106] and its targeted drugs of cetuximab had an antitumor effect [107]. The Hong Kong Cancer Institute performed an experiment for screening differentially expressed proteins between NPC cells with and without cetuximab [76]. The results showed that R-enolase, maspin, and p97 valosin-containing protein were up-regulated, while heat shock protein gp96 was down-regulated after cetuximab treatment. This approach is promising for illustrating the functional mechanisms of anticancer drugs. In the same year, to determine the functional components in signalling pathways as triggered by the Epstein-Barr virus-encoded latent membrane protein 1 (LMP1), Cao’s group combined the novel strategy of phosphoprotein enrichment with proteomics technology to illustrate the signalling cascade that is activated by LMP1 [77]. Because LMP1 could activate tumour-associated pathways, such as the NF-κB pathway, the differentially expressed proteins of CNE-1-LMP1 cells and CNE-1 cells via phosphate metal affinity chromatography (PMAC) were compared by MALDI-TOF-MS. These authors found that LMP1 could increase the quantity of total phosphoproteins, and 43 phosphoproteins showed significant changes when LMP1 was expressed. It is worth mentioning that this experiment is very interesting. Cao’s group planned to use phosphatase inhibitors for phosphoprotein enrichment and found that phosphatase inhibitors had an adverse effect on LMP1-mediated phosphorylation. Finally, the authors chose PMAC to enrich phosphoproteins *in vitro* [77].

In 2007, Yao’s group established 5-8F-GFP (green fluorescent protein) and 6-10B-GFP nasopharyngeal carcinoma cell lines [108] and demonstrated that highly metastatic 5-8F cells resulted in brain, cervical lymph node, and pulmonary metastases, while 6-10B cells were less metastatic in the nude mice model. Therefore, to screen proteins associated with NPC metastasis, 5-8F and 6-10B cells were subjected to 2-DE and MALDI-TOF-MS to identify differentially expressed proteins [78]. Fifteen differentially expressed proteins are listed in Table 1. These proteins are useful for studying the metastatic mechanism of NPC. For example, nm-23-H1 was highly expressed in highly metastatic cells 5-8F, and the down-regulation of nm-23-H1 by siRNA significantly increased the invasive ability of 6-10B [109]. Furthermore, nm-23-H1 down-regulation was positively correlated with lymph node and distant metastasis in primary NPC. In addition, patients with nm-23-H1 down-regulation in primary NPC had a poor prognosis.

In 2008, the differentially expressed proteins of an EBV-associated NPC cell line (C666-1) and a normal NP cell line (NP69) were analyzed by 2-DE and MALDI-TOF-MS [79]. Importantly, the down-regulated expression of annexin-II and β_2_-tubulin in C666-1 cells was also found to be lower in tumour cells than in adjacent normal epithelial cells of the NPC paraffin-embedded specimens [79], indicating that annexin-II and β_2_-tubulin might be potential targets for further NPC investigations.

During cancer development and progression, secretory proteins are related to intercellular communication, cell growth, adhesion, motility, and invasion [110,111]. Therefore, in 2009, CNE-2 cells were cultured with and without TGF-, and secretory proteins were obtained from conditioned serum-free media following 2-DE and MALDI-TOF-MS analyses known as secretome analysis [80]. Subsequently, amyloid β-protein precursor (APP) was up-regulated, and cystatin C was down-regulated with TGF-. These two proteins could be voided by the pretreatment of EGFR tyrosine kinase inhibitor, indicating that APP and cystatin C are EGFR-regulated secreted proteins in NPC cells. In addition, the positive expression of EGFR and APP and the negative expression of cystatin C were also found in NPC tissues [80]. A Hollow Fibre Culture System is a strategy for secretory protein enrichment [81]; to discover sensitive and specific biomarkers of NPC, Liao’s group used the Hollow Fibre Culture System combined with HPLC-MS/MS for secretome analysis and found that chloride intracellular channel protein 1 (CLIC1) of secretory protein was highly expressed in NPC surgically removed samples but rarely expressed in non-NPC tissues [81]. In addition, CLIC1 was used to discriminate plasma of NPC patients from healthy controls, finding that the sensitivity and specificity values were 63% and 77%, respectively [81]. These results indicate that CLIC1 could be applied as an early screening marker for NPC. In 2009, differentially expressed proteins of a cisplatin (CDDP)-resistant NPC cell line CNE-2-CDDP and parental CNE-2 cells were compared by 2-DE and MALDI-TOF-MS [82]. The results showed 13 proteins that were expressed highly in CNE-2-CDDP cells, including annexin-I. With immunohistochemistry, the up-regulation of annexin-I was associated with drug-resistance in NPC. However, whether this protein is related to drug-resistance is not clear, requiring further study.

Cancer cell secretome profiling is a promising strategy to search for biomarkers. Wu *et al.* [83] suggested that identified proteins were considered as potential marker candidates according to three strategies: (i) Identified proteins that are apparently secreted by one cancer type but not by others; (ii) Identified proteins that are released by most cancer cell lines; and (iii) Identified proteins that are putatively linked to cancer-relevant pathways. Subsequently, twenty-three cancer cell lines, including NPC, liver cancer, breast cancer cell line, *etc.*, of which the NPC cell line included NPC-TW02, NPC-TW04, and NPC-BM1, were subjected to secretome analysis by SDS and then LC-MS/MS [83]. Finally, cathepsin L1 and interferon-induced 17-kDa protein were considered potential serological cancer markers for NPC according to the above three strategies [83]. Another study about serum biomarkers was conducted. Chang *et al.*, [84] also used NPC-TW02, NPC-TW04, and NPC-BM1 to search for biomarkers, some of which, including cystatin A, manganese superoxide dismutase and matrix metalloproteinase 2, were indeed expressed at higher levels in NPC patients than in healthy controls and could be used to discriminate NPC patients from healthy persons with an AUC value for 0.83 in case–control study. To identify biomarkers that are related to the radioresistance of NPC, proteins from radioresistant subcloned cells (CNE-2-IR) and CNE-2 were compared by 2-DE and MALDI-TOF-MS [85]. The results showed that GRP78 and MnSOD were up-regulated, and 14-3-3σ and maspin were down-regulated in the radioresistant CNE-2-IR compared to CNE-2. For immunohistochemistry, 14-3-3σ and maspin were down-regulated, while GRP78 and Mn-SOD were up-regulated in CNE-2-IR compared to CNE-2. Radioresistant NPC patients were those with an incomplete regression of tumour with the completion of radiotherapy after six weeks or with recurrent disease at the nasopharynx and/or neck nodes with the completion of radiotherapy after two months [85,112]. In contrast, radiosensitive NPC patients were without the local residual lesions with the completion of radiotherapy after six weeks or recurrence with the completion of radiotherapy after two months [85,112]. In 2010, a study was conducted similar to Tang’s study [80,86]. However, unlike Tang’s study, Ruan *et al.* used TGF-α to stimulate CNE-2 cells for 10 min, while Tang *et al.* stimulated CNE-2 cells for 24 h [80,86]. As expected, the results with 10 min of 16 identified proteins were different from those with 24 h of 18 identified proteins, and annexin-3, keratin-8, and keratin-18 among the 16 proteins were validated as novel tyrosine-phosphorylation targets of EGFR signalling [86]. Phosphorylation and dephosphorylation are rapidly changing processes [113], and in this study, CNE-2 cells that were treated with TGF-α for 10 min that were subjected to secretome profiling immediately increased phosphorylated protein abundance compared to those that were treated for 24 h.

There were two other studies of proteomics in 2010. Prenylated Rab acceptor 1 (PRA1) is a binding partner for the EBV-encoded oncoprotein, LMP-1 [114]. Knowledge of the propensity of PRA would elucidate the nature of PRA1-LMP1 interaction and the tumourigenesis of NPC. Thus, proteins from PRA1-knockdown NPC cells and the control were analyzed by iTRAQ labelling approaches combined with LC-MS/MS, and the results showed that PRA1 was involved in lipid homeostasis and cell migration [87]. 14-3-3σ is a potential tumour suppressor and has been identified in many proteomics studies [72,73,75,78,84,85,87]. To explore the mechanism of 14-3-3σ in NPC invasion and metastasis, 14-3-3σ-associated proteins were identified by coimmunoprecipitation and ESI-TOF-MS [88]. 14-3-3σ, EGFR and keratin-8 interacted closely with NPC invasion and metastasis [88].

Because Tang *et al.* [80] and Ruan *et al.* [86] focused on studying EGFR pathways, many proteins were identified as EGFR-associated proteins. In 2011, 2-DIGE and MALDI-TOF-MS were used for phosphoproteins of the proteome in NPC CNE-2 cells with or without EGF stimulation [89]. As a result, two differential phosphoproteins (Glutathione *S*-transferase P1 and Growth factor receptor-bound protein 2) interacted with phospho-EGFR, and Glutathione *S*-transferase P1 was associated with the chemoresistance of paclitaxel. In 2011, to investigate the mechanisms of the p53-mediated radioresponse in NPC, proteins from p53 knockdown (CNE-2sip53) and a paired control were subjected to 2-DE and MALDI-TOF-MS, and the 14-3-3σ, prohibitin, annexin-A1, Glutathione *S*-transferase P1, proteasome subunit and keratin-18 of differential radioresponsive proteins were related to p53 protein [90]. In 2011, to search for EBV-associated proteins of NPC, proteins of NPC cells that were infected by EBV were compared with the control by iTRAQ-coupled 2-DE LC-MS/MS, and 12 proteins were significantly upregulated, associated with DNA binding, signal transduction, cytoskeleton formation, and metabolic pathways [91]. Some of these proteins, such as, Voltage-dependent anion-selective channel protein 1, High mobility group protein B1 and ubiquitin were linked to the p53-mediated signalling pathway [91]. In addition, Dynein, tubulin and S100-A2 may participate in the NF-κB pathways [91]. S-18 is a subclone of CNE-2 and possesses the greatest migration and invasion ability [115]. In 2011, differentially expressed proteins of S-18 and CNE-2 cells were detected by 2-DE and MALDI-TOF MS, and HSP27 and ezrin were up-regulated, while valosin-containing protein and keratin-18 were down-regulated in S-18 [11]. Importantly, HSP27 enhanced the metastatic property of NPC cells probably via the NF-κB-mediated activation of matrix metalloproteinases (MMPs).

In 2012, differentially expressed proteins of radioresistant CNE-1 cells (CNE-1-IR) and CNE-1 cells were detected by 2-DE and MALDI-TOF-MS, and the expression of HSP27 was significantly higher in CNE-1-IR than in CNE-1 cells [92]. In addition, CNE-1-IR cells that were treated with an inhibitor of HSP27 showed high sensitivity to radiation [92], demonstrating that HSP27 is a radioresistance-related protein in NPC cells. Currently, subcellular proteomics, such as organelles, would more effectively discover functionally related proteins mainly because the subcellular proteome is much simpler than the whole cell proteome and can maximally identify the protein components in a subcellular proteome. Thus, mitochondrial differentially expressed proteins of metastatic (5-8F) and non-metastatic (6-10B) cells were analyzed by 2D-DIGE and MS [93]. The results showed that mitochondrial differentially expressed proteins, including peroxiredoxin 3, peroxiredoxin 6, superoxide dismutase, δ(3.5)-δ(2.4)-dienovl-CoA isomerase, maspin, cytochrome c oxidase subunit 5A, mitochondrial precursor, protein disulfide isomerase related protein 5, eukaryotic initiation factor 5A, isocitrate dehydrogenase, and 26S protease regulatory subunit 7 isoform 1, have been detected in the tumour-stroma co-evolution model in that mitochondrial oxidative stress contributes to tumour metastasis. In addition, peroxiredoxin 3 was highly expressed in 5-8F, and its suppression showed an increased mobility potential in 5-8F [93]. Another study about differentially expressed proteins was conducted in 2012. Because NESG1 is a tumour-suppressor gene that inhibits cell proliferation, invasion and migration of NPC cells [116], proteins of NESG1-overexpressing NPC 2F4, 3D8 cells and their control of C6 cells were subjected to 2-DE and MALDI-TOF-TOF [94]. Twenty-six differentially expressed proteins were found, and enolase 1 (α) was a negatively regulated gene of NESG1 in NPC. In 2012, cells that were resistant to *cis*-diamminedichloroplatinum (CNE-2/cDDP) and CNE-2 were screened for differentially expressed proteins by 2-DE and ESI-Q-TOF-MS. The results showed that Keratin-1, cathepsin D and annexin-A5 were highly expressed in CNE-2/cDDP compared to CNE-2 [95]. Furthermore, the suppression of Keratin-1 expression by siRNA resulted in decreasing multidrug resistance in siRNA-CNE-2/cDDP cells. In 2012, no previous study had examined the inflammasome components in NPC tumour cells. Thus, proteins from Apoptosis-associated speck-like protein containing a caspase recruitment domain expressing HK1 (ASC-expressing HK1) cells were treated with EBV noncoding RNA, H_2_O_2_, and poly (dA:dT) to stimulate NLRP3, AIM2, and RIG-I inflammasomes, respectively, and the controls were compared by iTRAQ-coupled LC-MS/MS [96]. Subsequently, end-binding protein 1 was upregulated in stimulating HK1/ASC cells that served as a crucial component for the speck-like particle formation of activation in the absence in melanoma 2 inflammasomes [96]. Salt fish is an important source of nitrosamines and is carcinogenic [97]. N, N0-Dinitrosopiperazine (DNP) is a predominant volatile nitrosamine in salted fish that not only induces NPC but also facilitates NPC metastasis [117]. Thus, to investigate the mechanism of DNP-mediated NPC metastasis, proteins from 6-10B cells (low metastasis) that were treated with DNP and the control were analyzed by SILAC and LC-MS/MS. SILAC profiling relied on living cells of uniformly and specifically labelling. Finally, the results showed that DNP may regulate cytoskeletal, anterior gradient-2, cathepsin, and clusterin proteins were involved NPC metastasis.

Our group performed a study related to radioresistance-associated proteins in 2013. Proteins from CNE-2R and its parental cell line CNE-2 were analyzed by 2-DE and MALDI-TOF-MS [99]. The protein expression that varied significantly between the two cell lines is displayed in Table 1. Today, low-molecular-mass proteins secretome profiling represents an attractive pool for cancer biomarker discovery [100]. Therefore, to seek potential plasma biomarkers and therapeutic targets for NPC, conditioned media of NPC-TW02, NPC-TW04, and NPC-BM1 cells were used with tricine-SDS-gel-assisted fractionation in conjunction with LC-MS/MS [100]. As a result, 35 low-molecular-mass proteins (<15 kDa) were identified as candidate NPC biomarkers, and the plasma C–C motif chemokine 5 showed good power (AUC 0.801) in discriminating NPC patients from healthy controls. In 2013, because isodeoxyelephantopin had antitumor effects against NPC, CNE-1 cells that were treated with isodeoxyelephantopin or not were subjected to SILAC and MS to explore the underlying mechanism of antitumor effects [101]. The results showed that isodeoxyelephantopin induced the antitumor inflammation factor pathway and G2/M arrest via ROS-dependent DNA damage and mitochondria-mediated apoptosis pathways. In 2013, cripto-1, overexpressed in a wide range of epithelial carcinomas, was reported to play an important role during tumorigenesis [118]. Interestingly, the expression of cripto-1 is also significantly increased in NPC [119]. Thus, to investigate the NPC clinicopathological correlations of cripto-1, proteins from cripto-1 over-expressed cells (CNE-1/CR1^+^) and parental cells were analyzed by 2D-DIGE MALDI-TOF MS [102]. As a result, 23 differentially expressed proteins, including 14-3-3γ, were found, and the high level of 14-3-3γ was associated with lymph node metastasis, distant metastasis and clinical stages. Thus, 14-3-3γ might be a biomarker for the prognosis of patients with NPC.

In 2014, 6-10B treated with DNP and controls were used for SILAC and LC–MS/MS for the quantitative proteomics of phosphoproteins, of which TiO_2_ was used for phosphopeptide enrichment [103]. As a result, 48 phosphorylation sites on 30 unique phosphopeptides were mediated by DNP. In addition, DNP induced lysine-rich CEACAM1 co-isolated protein (LYRIC) phosphorylation at serine 568 to facilitate cell motility and invasion in both 6-10B and CNE-1 cells.

### 3.2. Proteomics Research in Xenograft Mice Models Related to Nasopharyngeal Carcinoma

Compared to cancer patients with tumors, animal models used to screen for markers related to cancer provide advantages such as: (i) Complexity and variability of serum proteins in different patients, making data interpretation much more difficult; and (ii) The lack of tumour-free controls serum from the same patients, making the use of human sera to explore the tumour markers unpractical [120]. In fact, the most feasible system is the tumour xenotransplantation model in nude mice.

In 2004, the plasma proteins of healthy nude mice and nude mice that were inoculated with one of five human cancer cell lines (stomach adenocarcinoma, HONE-1, colon adenocarcinoma, oral cancer, and glioblastoma multiform) were compared by 2-DE, MALDI-TOF MS, and LC-MS/MS [120]. Subsequently, the mouse haptoglobin β-subunit was up-regulated in tumour-bearing mice of five cell lines, while the mouse haptoglobin α-subunit was up-regulated in colon adenocarcinoma, nasopharyngeal carcinoma, and oral cancer (Table 2). Haptoglobin is an acute-phase protein (APP), a circulating plasma protein that is induced by the early and nonspecific innate immune response [121,122]. In addition, a panel of APPs might serve as screening biomarkers of NPC for early cancer detection.

**Table 2:** The proteomics of mice xenotransplant models as related to nasopharyngeal carcinoma.

Year	Proteins	Description	Reference
2004	Haptoglobin β-subunit and haptoglobin α-subunit were up-regulated in nude mice that were inoculated with five human cancer cell lines.	Nude mice inoculated with one of five human cancer cell lines: stomach adenocarcinoma, HONE-1, colon adenocarcinoma, oral cancer, and glioblastoma multiform *vs.* healthy nude mice; 2-DE, MALDI-TOF MS, and LC-MS/MS.	[120]
2008	Peroxiredoxin 2, *etc.*, were up-regulated in NPC xenograft model with NPC-TW02 cells.	NPC xenograft model with NPC-TW02 cells *vs.* the control; 2D-DIGE and MALDI-TOF-MS.	[123]
2008	HSP70 and mucin 5B were up-regulated in the NPC of mice that were induced by DNP.	NPC of mice that were induced by DNP *vs.* normal nasopharynx; 2-DE and MALDI-TOF-MS.	[124]

In 2008, proteins from serum samples of NPC-bearing and control mice were used for 2D-DIGE and MALDI-TOF-MS [123]. Peroxiredoxin 2 and carbonic anhydrase 2 were elevated in the xenograft mouse model compared to controls [123]. In addition, Western blot analysis confirmed that Prx-II and CA-II were up-regulated in plasma from five NPC patients, and ELISA analysis showed that Prx-II was significantly higher in plasma of NPC patients, in agreement with the result of MS. Thus, Prx-II might be a potential NPC biomarker. In 2008, the carcinogen DNP was also studied in xenograft mice models as related to NPC [124]. Proteins that were extracted from the NPC of mice that were induced by DNP and those with a normal nasopharynx were analyzed by 2-DE and MALDI-TOF-MS. As a result, HSP70 and mucin 5B were up-regulated in mouse NPC and atypical hyperplasia nasopharyngeal tissues [124]. Furthermore, HSP70 and mucin 5B were expressed in a dose-dependent manner in normal nasopharyngeal epithelia cells that were treated with DNP, indicating that HSP70 and mucin 5B might be important targets in nasopharyngeal tumorigenesis induced by DNP. However, studies of in xenograft mice models that were related to nasopharyngeal carcinoma have disappeared in recent years. This phenomenon may due to the new identification techniques of proteomics that have gradually appeared, such as SELDI-TOF-MS. In addition, xenograft mice models were used for quantitative proteomics, resulting in many proteins being related to invalid proteins that come from mice. Therefore, the direct analysis of serum proteins from cancer patients gradually became the popular method.

### 3.3. Proteomics Research in Human Tissue or Fluid of NPC

The proteomics research in human tissue or fluid of NPC dates back to 2005. Advanced NPCs can be divided into three clinical types: cranial type A, generalized cervical lymphadenopathy type D, and mixed type AD [125]. In fact, these different types have different biological behaviours and deserve different therapeutic plans. Serological testing has many advantages, such as easily collecting samples and noninvasive testing. Therefore, the serum samples of 29 type A and type D were used for SELDI-TOF-MS analysis [7]. The results showed that there were 11 differentially expressed proteins between the two types, including *m*/*z* 4053 Da, *m*/*z* 4207 Da, and *m*/*z* 5901 Da, which were highly expressed in type A (Table 3). For peak reproducibility, a multi-layer artificial neural network with a scaled conjugate gradient optimized the back propagation algorithm to discriminate type A from type D. Pattern recognition techniques have been applied to diverse areas, including mass spectrometry [126]. Finally, this artificial neural network could distinguish type A from type D NPC patients with 89.5% accuracy, indicating that the identified proteins were credible. In 2005, sera proteins from 10 NPC patients and 10 controls were used for 2-DE and MALDI-TOF-MS [127]. As a result, ceruloplasmin was highly expressed in NPC serum. For ELISA and immunohistochemical analyses, ceruloplasmin was also highly expressed in NPC patients compared to healthy controls. Interestingly, in NPC patients six months after efficient treatment, the expression of ceruloplasmin had vanished, ruling out ceruloplasmin as a biomarker for the diagnosis and monitoring of NPC.

In 2007, there were three studies of serological markers. One of these studies was of the humoral immune response. Sera proteins from 19 NPC patients and 19 controls were analyzed by 2-DE, MALDI-TOF-MS, and ESI-Q-TOF-MS [128]. The identified proteins of keratin-19, Erb3-binding protein, and Rho GDP dissociation inhibitor-β further confirmed that triplets of them were expressed significantly higher in NPC patients than in other types of cancer patients and healthy persons. These results indicate that keratin-19, Erb3-binding protein, and Rho GDP dissociation inhibitor-β might have specific utility in NPC screening and diagnosis. Because SELDI-TOF-MS has many advantages, such as being fast, high-throughput, allowing lower amounts, and resolving low-mass proteins, it is directly applicable for developing clinical assays [129]. Another study used SELDI-TOF-MS to analyze serum samples from NPC patients and healthy controls [130]. Four of the identified protein peaks at 4097, 4180, 5912, and 8295 Da were used for peak reproducibility for decision tree classification by Biomarker Pattern Software, and cross-validation sensitivity and specificity were considered as the tree’s discriminative power. Surprisingly, the results showed that the sensitivity and specificity were 92% and 92.9%, respectively. It seems that the serum protein profiling approach of SELDI-TOF-MS combined with a tree analysis model can efficiently discriminate NPC from non-cancer persons. A reference map of the NPC proteome was established by Chen’s group in 2007 [131]. Protein extractions from 20 fresh NPC biopsy specimens were analyzed by 2-DE, MALDI-TOF-MS and ESI-Q-TOF-MS. There were 216 identified proteins that can be accessed online (http://www.xyproteomics.org/). These proteins can be used for comparison among different pathological types of NPC, different stages in the process of NPC, or between the NPC plasma/tissue and normal nasopharyngeal epithelial plasma/tissue.

Using tissue samples from patients may be the most direct way to find biomarkers for cancers via a proteomic approach. However, because tumour specimens provide tissue heterogeneity with different infiltrating lymphocytes and stroma, several methods have been used to obtain homogeneous cell populations from a heterogeneous tissue, for example, laser capture microdissection (LCM) [132]. In 2008, proteins from NPC and normal nasopharyngeal epithelial tissues by LCM were subjected to 2-DE and MALDI-TOF-MS and ESI-QTOF-MS [133]. Subsequently, 36 differentially expressed proteins between the NPC and normal nasopharyngeal epithelial tissues were identified and are displayed in Table 3. Among them, stathmin, 14-3-3σ, and annexin-I in NPC tissues were related to the differentiation degree and/or metastatic ability of the NPC cell lines [133]. In fact, patients with cathepsin D up-regulation had a poor prognosis, and the down-regulated expression of cathepsin D by siRNA significantly decreased the invasive ability of 5-8F cells *in vitro* [132]. In addition, keratin-18 was down-regulated in NPC *vs.* normal nasopharyngeal epithelial tissues but was up-regulated in lymph node metastasis *vs.* primary NPC. In addition, patients with keratin-18 up-regulation had a poor prognosis [23]. Consequently, cathepsin D and keratin-18 are potential biomarkers for the prognosis of NPC. In 2008, to identify novel proteins that were related to the pathogenesis of NPC, protein extractions from NPC and adjacent noncancerous nasopharyngeal epithelial tissue were analyzed by 2-DE, MALDI-TOF-MS, and ESI-Q-TOF-MS [134]. As a result, 21 identified proteins were differentially expressed between NPC and adjacent noncancerous nasopharyngeal epithelial tissue. Importantly, one of those proteins was Raf kinase inhibitor protein, which is a potential metastasis suppressor [135]. Furthermore, Raf kinase inhibitor protein was significantly down-regulated in high-metastatic 5-8F cells compared to low-metastatic 6-10B cells, suggesting that Raf kinase inhibitor protein may be an NPC metastasis suppressor. A panel of differentially expressed proteins that were related to NPC metastases were also detected in 2008. Serum samples from NPC patients with lymph node metastases and controls were subjected to 2-DE and MALDI-TOF-MS [136]. The identified proteins are shown in Table 3.

**Table 3:** The proteomics of human body fluid or tissue related to nasopharyngeal carcinoma.

Year	Proteins	Description	Reference
2005	*m*/*z* 4053 Da, *m*/*z* 4207 Da, and *m*/*z* 5901 Da were up-regulated in type A NPC.	Type A of NPC *vs.* type D of NPC; SELDI-TOF-MS.	[7]
2005	Ceruloplasmin was up-regulated in NPC patients.	NPC patients *vs.* healthy persons; 2-DE and MALDI-TOF-MS.	[127]
2007	HSP 70, HSP 60, Prohibitin, keratin-19, *etc.*, were up-regulated in NPC patients.	NPC patients *vs.* Healthy persons; 2-DE, MALDI-TOF-MS, and ESI-Q-TOF-MS.	[128]
2007	*m*/*z* 8295 Da and *m*/*z* 4180 Da were up-regulated in NPC patients; *m*/*z* 5912 Da and *m*/*z* 4097 Da were down-regulated in NPC patients.	NPC patients *vs.* Healthy persons; SELDI-TOF-MS.	[130]
2008	Keratin-5, nm-23 protein, vimentin, HSP 27, and stathmin were up-regulated in NPC; Cathepsin D, annexin-I, keratin-8, 14-3-3σ, and keratin-18 were down-regulated in NPC.	NPC *vs.* normal nasopharyngeal epithelial tissues; 2-DE and MALDI-TOF-MS and ESI-Q-TOF-MS.	[133]
2008	Keratin-8, keratin-19, α-enolase, annexin-A3, annexin-A1, and HSP27 were up-regulated in NPC; Vimentin, triosephosphate isomerase, superoxide dismutase, prohibitin, and Raf kinase inhibitor protein were down-regulated in NPC.	NPC *vs.* adjacent noncancerous nasopharyngeal epithelial tissue; 2-DE, MALDI-TOF-MS, and ESI-Q-TOF-MS.	[134]
2008	HSP27, slCAM-l, cathepsin G, *etc.*, were up-regulated in NPC patients with lymph node metastasis; NM-23-H1 proteins, *etc.*, were down-regulated in NPC patients with lymph node metastasis.	NPC patients with lymph node metastasis *vs.* the controls; 2-DE and MALDI-TOF-MS.	[136]
2009	Keratin-31, vimentin, prohibitin, *etc.*, were up-regulated in tumour stroma; Rho GDP dissociation inhibitor (GDI) β, superoxide dismutase, keratin-19, and annexin-A2 were down-regulated in tumour stroma.	Tumour stroma *vs.* normal stroma; 2-DE, and MALDI-TOF-MS/ESI-QTOF-MS.	[137]
2009	Periostin, keratin-1, nm-23 protein, *etc.*, were up-regulated in tumour stroma; HSP 70, keratin-19, Rho GDP dissociation inhibitor (GDI) β, superoxide dismutase, *etc.*, were down-regulated in tumour stroma.	Tumour stroma *vs*. normal stroma; 2-DIGE, and MALDI-TOF-MS/ESI-QTOF-MS.	[34]
2009	Heterogeneous nuclear ribonucleoprotein Q, Superoxide dismutase mitochondria, *etc.*, were up-regulated in NPC tissues; Vimentin, α-enolase, heterogeneous nuclear ribonucleoproteins C1/C2 Raf kinase inhibitor protein, *etc.*, were down-regulated in NPC tissues.	NPC tissues *vs.* normal nasopharyngeal epithelial tissues; 2-DE and ESI-Q-TOF-MS.	[138]
2009	*m*/*z* 13,738.6 protein was up-regulated in NPC; *m*/*z* 3159.835 and 5187.656 proteins were down-regulated in NPC.	NPC *vs.* non-cancer; SELDI-TOF-MS.	[139]
2010	*m*/*z* 8605, 2791, and 7154 were up-regulated in NPC patients; *m*/*z* 5320, 5355, 5380, 5336 and 9366 were down-regulated in NPC patients.	NPC patients *vs.* Controls; SELDI-TOF-MS.	[140]
2010	Galectin-1 was up-regulated in NPC tissues.	NPC tissues *vs.* normal nasopharyngeal epithelial tissues; 2-DE and MALDI-TOF-MS.	[141]
2010	Stathmin 1 was up-regulated in NPC tissues; Cathepsin D, keratin-8, and 14-3-3σ were down-regulated in NPC tissues.	NPC tissues *vs.* normal nasopharyngeal epithelial tissues; iTRAQ and LC-MS/MS.	[142]
2011	*m*/*z* 2019.691, 2223.114, 2244.074, 2467.500, 2491.888, 7977.352, *etc.*, were up-regulated in NPC patients.	NPC patients *vs.* the controls; SELDI-TOF-MS.	[143]
2011	*m*/*z* 808.99 and 834.61 were up-regulated in NPC patients.	NPC patients *vs.* the controls; MALDI-TOF-MS.	[144]
2011	KIT, ATP1A1, synapsin, SEK1, and histone H2AX were up-regulated in Relapsed NPC; C-Jun was down-regulated in Relapsed NPC.	Relapsed NPC *vs.* primary NPC; Protein array analysis.	[145]
2011	Kallicrein and thrombin-antithrombin III complex were up-regulated in NPC patient.	NPC patients *vs.* Healthy controls; 2-DIGE and LC–MS/MS.	[146]
2012	*m*/*z* 4155, 4194, 4210, and 4249 were up-regulated in NPC with liver metastasis.	NPC with liver metastasis *vs.* NPC without liver metastasis; MALDI-TOF-MS.	[147]
2012	*m*/*z* 1051.37 and 1262.67 were up-regulated in NPC patients; *m*/*z* 3193.14 and 4055.47 were down-regulated in NPC patients.	NPC patients *vs.* healthy controls; MALDI-TOF-MS.	[148]
2012	Manganese superoxide dismutase (Mn-SOD), HSP 27, Glutathione *S*-transferase ω1 (GST ω1), *etc.*, were up-regulated in Radioresistant NPC tissues; Prohibitin, *etc.*, were down-regulated in Radioresistant NPC tissues.	Radioresistant NPC tissues *vs.* Radiosensitive NPC tissues; 2-DE and MALDI-TOF-MS.	[149]
2012	Nm-23 protein, periostin, and keratin-1 were up-regulated in the stroma of NPC; Superoxide dismutase, Rho GDP dissociation inhibitor (GDI) β, keratin-19, and HSP 70 were down-regulated in stroma of NPC.	Stroma of NPC *vs.* stroma of normal nasopharyngeal mucosa; LCM, 2D-DIGE, MALDI-TOF-MS, and ESI-Q-TOF-MS.	[150]
2013	*m*/*z* 2931, 3474, *etc.*, were up-regulated in radiation-sensitive patients; *m*/*z* 2873, 4098, *etc.*, were down-regulated in radiation-sensitive patients.	Radiation sensitive patients *vs.* radiation resistant patients; SELDI-TOF-MS.	[151]
2014	Cyclophilin A (CYPA) was up-regulated in NPC biopsies; Enolase-1 (ENO1) was down-regulated in NPC biopsies.	NPC biopsies *vs.* normal nasopharyngeal epithelial tissues; LCM, 2D-DIGE and MALDI-TOF-MS.	[152]

In 2009, proteins from the stroma of NPC and normal nasopharyngeal epithelium tissues were assessed by LCM, 2-DE, and MALDI-TOF-MS/ ESI-QTOF-MS [137]. Sixty differential proteins were identified, and CapG was upregulated in the stroma cells of NPC compared to those of the control. In fact, CapG was a tumour promoter that modulated the invasive properties of cells during tumorigenesis [153,154]. CapG might be a target for NPC treatment, requiring further study. Similarly, another study of the stroma was conducted in the same way using 2-DIGE instead of 2-DE [34]. However, the latter identified only 20 different proteins. The differentially expressed proteins are listed in Table 3. There were some identical proteins in the two studies, such as Rho GDP dissociation inhibitor (GDI) β, Superoxide dismutase, and Keratin-19. In 2009, proteins from NPC and normal nasopharyngeal epithelial tissues were used to screen differentially tyrosine phosphorylated proteins by 2-DE and ESI-Q-TOF-MS [138]. A total of 13 tyrosine phosphorylated proteins were identified, including seven up-regulated and six down-regulated proteins in NPC compared to normal nasopharyngeal epithelial tissues as listed in Table 3. In 2009, SELDI-TOF-MS was used for proteomic analysis as directly related to NPC biomarkers. Serum proteins from NPC patients and control subjects without cancer were analyzed [139]. As a result, *m*/*z* 3159.835 and 5187.656 proteins showed low expression, and *m*/*z* 13,738.6 protein was highly expressed in NPC compared to the controls, with a sensitivity of 91.66% and a specificity of 95.83% by the Decision Tree model.

In 2010, SELDI-TOF-MS was also used directly for serum proteins from NPC patients and controls. After proteomics analysis, a training test was performed [140]. As a result, eight proteomic biomarkers were selected as the potential biomarkers of NPC with a sensitivity of 90.9% and specificity of 92.0% (Table 3), which was better than the diagnostic method using the EBV capsid antigen IgA antibodies (VCA-IgA). In 2010, another two studies were performed to analyze aberrantly expressed proteins in NPC tissues. One study showed that Galectin-1 was approved as a potential diagnostic marker or therapeutic target for NPC by MALDI-TOF-MS, RT-PCR, and Western blot between NPC and normal nasopharyngeal epithelial tissues [141], which was also highly expressed in CNE-2R cells [99]. For the other study, the expression of the four proteins of stathmin 1, cathepsin D, keratin-8, and 14-3-3σ was considered to correlate with the differentiation of NPC histological types [142].

In 2011, to screen for biomarkers in patients with NPC, serum proteins from NPC patients and healthy persons were subjected to SELDI-TOF-MS [143]. Nine proteins were highly expressed in NPC compared to in the controls (Table 3). However, a decision tree classification or a training test was not performed in this study, and the results presented low persuasion. Similarly, serum proteins from NPC patients and the controls were used for MALDI-TOF-MS to screen biomarkers in 2011 [144]. After quantitative proteomics and blind testing, four proteins of *m*/*z* 808.99, 834.61, 3954.82 and 8141.88 were used for a diagnostic model with a sensitivity of 80.0% and a specificity of 60.4% [144]. In addition, another two serum proteins of the Kallicrein and thrombin-antithrombin III complex were up-regulated in NPC patients and may serve as potential biomarkers [146]. In 2011, to analyze the aberrantly expressed phosphorylated proteins in relapsed NPC, proteins from relapsed NPC and primary NPC tissues were subjected to protein array analysis, and KIT, ATP1A1, Synapsin, SEK1 and histone H2AX were up-regulated in relapsed NPC, while c-Jun was down-regulated [145]. However, our previous research indicated that the expression of c-Jun was up-regulated in radioresistant CNE-2R cells compared to CNE-2 cells, and the shRNA-mediated knockdown of c-Jun in the CNE-2R cells enhanced the radiosensitivity of CNE-2R cells [155,156]. Thus, the aberrantly expressed c-Jun may be a target for NPC treatment. 

NPC usually metastasizes to the lung, bone and liver, and the prognosis is often poor. To investigate whether the behaviour of liver metastasis is associated with changes in the plasma proteins of NPC, serum proteins from NPC with liver metastasis and NPC without liver metastasis were analyzed by MALDI-TOF-MS [147]. As a result, four proteins that were highly expressed in NPC patients with liver metastasis as displayed in Table 3 were useful diagnostic markers for the existence of liver metastasis in NPC [147]. In 2012, MALDI-TOF-MS was used to seek biomarkers for the early diagnosis of NPC. In addition, four proteins (Table 3) were selected to train a genetic algorithm model, resulting in a sensitivity of 100% and a specificity of 100% [148]. According to the principles introduced above [78,109], proteins from radioresistant and radiosensitive NPC tissues were subjected to 2-DE and MALDI-TOF-MS to screen biomarkers to predict the nasopharyngeal carcinoma response to radiotherapy [149]. Among the biomarkers, ERp29 was approved as the biomarker with the highest potential for predicting NPC response to radiotherapy. In 2012, to screen stroma-associated proteins that are involved in NPC carcinogenesis, proteins from stroma of NPC and normal nasopharyngeal mucosa were subjected to quantitative proteomic analysis [150]. The differentially expressed proteins are displayed in Table 3. Among these proteins, periostin was used for further study, and the over-expression of periostin was associated with advanced clinical stage lymph node metastasis and decreased overall survival in NPC [150].

In 2013, our research group screened serum biomarkers to predict the radiosensitivity of NPC. The serum proteins of pre-and post-treatment NPC patients were analyzed, and 11 protein peaks were significantly different [151]. Among these proteins, 4 proteins of *m*/*z* 2575, 3942, 6117, and 6778 were used for classification for tree construction with 78.0% diagnostic accuracy [151].

In 2014, the expression levels of Enolase-1 (ENO1) and Cyclophilin A (CYPA) in NPC were significantly correlated with the clinical stage via quantitative proteomics and immunohistochemistry [152].

Identical proteins are sorted in Table 1, Table 2 and Table 3: Keratin is ranked the highest followed by annexin, heat shock protein, 14-3-3σ, nm-23 protein, cathepsin, heterogeneous nuclear ribonucleoproteins, enolase, triosephosphate isomerase, stathmin, prohibitin, and vimentin. Keratin is a protein related to invasion, metastasis, multidrug resistance, and it is involved in the EGFR signalling pathway of NPC [86,88,95]. Keratin is expressed significantly higher in NPC patients than in healthy persons which is correlated with the differentiation of NPC histological types [128,142]. Besides, Keratin expression levels correlate with tumor characteristics and clinical outcome in metastatic breast cancer and Keratin serves as a cancer stem cell marker of the squamous cell carcinoma [157,158]. Annexin is a calcium/phospholipid-binding protein related to invasion, metastasis, multidrug resistance, p53 protein function, and it is involved in the EGFR signalling pathway of NPC [82,86,90,95,133]. Its overexpression is responsible for poor prognosis in basal like breast cancer, gastrointestinal stromal tumor, and colorectal cancer [159,160,161,162]. The role of heat shock protein in carcinogenesis is still uncertain, as heat shock protein β-1 protein is expressed at low levels in drug-resistant NPC cells, whereas heat shock protein 90 is highly expressed in drug-resistant NPC and colorectal cancer cells [95,163]. In this review, heat shock protein 27 is related to metastasis, radioresistance, and tumorigenesis of NPC [11,92,124,136]. In other cancers, heat shock protein 70 is overexpressed in breast cancer, associated with cancer progression, and heat shock protein gp96-positive expression is correlated with poor survival in gallbladder cancer [164,165].

14-3-3σ is an adapter protein which is implicated in the regulation of a large spectrum of both general and specialized signaling pathways. It binds to a large number of partners, usually by recognition of a phosphoserine or phosphothreonine motif and may also regulate E3 ubiquitin-protein ligase Mdm2 (MDM2) auto-ubiquitination and degradation and thereby activate p53/TP53 [166]. In this review, 14-3-3σ is related to p53 function, invasion, metastasis, multidrug resistance, and differentiation degree of NPC [75,88,90,95,142]. It is a potential tumour suppressor and has been identified in many proteomics studies [72,73,75,78,84,85,87]. Nm-23 protein is originally identified as a metastasis suppressor protein [167]. It is highly expressed in highly metastatic NPC cells, and the down-regulation of nm-23 increased the invasive ability of NPC cells [109]. Besides, nm-23 is involved in gastric carcinoma pathogenesis and nm-23-positive patients have a good prognosis compared to the nm-23-negative patients of breast cancer [167,168]. Cathepsin is considered as a potential serological cancer marker for NPC [83], and is considered to correlate with the differentiation, multidrug resistance, and metastasis of NPC [95,117,142]. NPC and melanoma patients with cathepsin up-regulation have a poor prognosis [133,169]. Actually, it is often thought to act as a tumor promoter which is involved in the degradation of extracellular matrix, enhancing tumor progression and metastasis [170].

Heterogeneous nuclear ribonucleoproteins (hnRNP) complexes provide the substrates for the processing events that pre-mRNAs undergo before becoming functional, translatable mRNAs in the cytoplasm [171]. The mechanism for the up-regulation of heterogeneous nuclear ribonucleoprotein A3 in CNE-1-IR cells, while heterogeneous nuclear ribonucleoprotein A1 is down-regulated, remains unclear [92]. Recent research shows that heterogeneous nuclear ribonucleoproteins inhibit endometrial cancer [172]. Further, heterogeneous nuclear ribonucleoprotein is a marker of transitional cell carcinoma and colorectal cancer [173,174]. Enolase, in addition to its role in glycolysis, plays a part in various processes such as growth control, hypoxia tolerance and allergic responses. It is involved in regulation of the NESG1 gene that inhibits cell proliferation, invasion and migration of NPC [116]. In other cancers, enolase can be a potential prognostic marker, since high expression of enolase is associated with high migration and invasion properties in glioma carcinoma and non-small cell lung cancer cells [175,176]. Triosephosphate isomerase is a highly conserved component of glycolysis. It is commonly detected in NPC, given that tumors are always dependent on glycolysis for energy and metabolite production [177].

Stathmin participates in growth arrest and apoptosis of NPC cells, and is related to the degree of differentiation in NPC tissues [72,73,133,142]. It is up-regulated in CNE-2R cells in relation to radioresistance [99]. Moreover, stathmin is involved in the resistance to taxol among different cancer cell lines [178]. Thus, stathmin may have therapeutic potency for decreasing radioresistance and drug-resistance in cancers. Prohibitin is up-regulated in sera and tumour stroma while down-regulated in NPC tissues compared to healthy controls [128,134,137,149]. Furthermore, prohibitin protects against colitis-associated cancer by modulating p53-mediated apoptosis in NPC [90,179]. Vimentin is a class-III intermediate filament that is involved in a programmed cell death process [180]; it is controversial whether vimentin is up-regulalted or down-regulated according to previous studies of NPC [133,134,137,138]. One study demonstrated that the expression levels of vimentin were significantly up-regulated in renal cell cancer tissues compared to adjacent non-cancerous tissues, which could enhance migration and invasion activities of cancer cells [181]. These findings suggest that vimentin is a potential therapeutic target in cancer.

## 4. Conclusions

Proteomics is widely used in NPC to search for biomarkers and compare differentially expressed proteins. This study presents an overview of proteomics with different samples related to NPC described in chronological sequence. This study also shows the common methods that are widely used for NPC proteomics. Keratin is the protein ranked most highly in these proteomic studies of NPC, followed by such proteins as annexin, heat shock protein, 14-3-3σ, nm-23 protein, cathepsin, heterogeneous nuclear ribonucleoproteins, enolase, triosephosphate isomerase, stathmin, prohibitin, and vimentin, indicating that these proteins may be NPC-related proteins and have potential value for further study.
